# Building-level wastewater surveillance localizes interseasonal influenza variation

**DOI:** 10.1128/msphere.00600-23

**Published:** 2024-01-03

**Authors:** Emma R. Germano, Tiffany Flores, Grace S. Freed, Kang Kim, Grace H. Tulinsky, Annie Yang, Oliver J. Rose, Caroline A. Ray, April Autry, Marina Catallozzi, Brian J. Mailloux, JJ L. Miranda

**Affiliations:** 1 Department of Biology, Barnard College, Columbia University, New York, New York, USA; 2 Environmental Science Department, Barnard College, Columbia University, New York, New York, USA; 3 Office of Facilities Services, Barnard College, Columbia University, New York, New York, USA; 4 Office of Health and Wellness, Barnard College, Columbia University, New York, New York, USA; 5 Heilbrunn Department of Population and Family Health, Mailman School of Public Health, Columbia University, New York, New York, USA; 6 Department of Pediatrics, Columbia University Irving Medical Center, New York, New York, USA; University of Michigan, Ann Arbor, Michigan, USA

**Keywords:** influenza A virus, wastewater, wastewater-based epidemiological monitoring, New York City, students

## Abstract

Influenza virus poses a recurring threat to public health and infects many populations in annual waves of generally unpredictable magnitude and timing. We aimed to detect the arrival and estimate the case magnitude of seasonal influenza A in urban New York City college dormitory buildings. Our wastewater-based surveillance (WBS) program measured viral RNA in the sewage outflow of three dormitories at Barnard College in 2021 and 2022. Wastewater test positivity strongly correlated with New York County clinical cases (Kendall’s τ = 0.58). Positive wastewater samples are also associated with campus clinical cases. The 2022 data stand in stark contrast to the 2021 results by revealing the more frequent and earlier presence of influenza A. The increase in positive tests is significant (*P* < 0.01). It is further noteworthy that positive samples were not evenly distributed among buildings. Surveillance additionally identified the influenza A H3 subtype but did not detect any influenza B. We also systematically analyzed our viral purification protocol to identify in which fraction influenza can be found. While virus can be found in solid fractions, a substantial quantity remains in the final liquid fraction. Our work focuses on individual buildings rather than larger sewersheds because buildings may localize interseasonal influenza variation to specific subpopulations. Our results highlight the potential value of building-level WBS in measuring influenza incidence to help guide public health intervention.

**IMPORTANCE:**

Seasonal influenza remains a major public health burden. We monitored influenza A in dormitory wastewater of a New York City college in 2021 and 2022. Longitudinal samples acquired over consecutive years allowed measurement of individual buildings between seasons. We uncovered building-level changes in the magnitude and timing of test positivity concordant with clinical cases. Surveillance also localized the heterogeneity of influenza variation during the large 2022 seasonal surge. The ability to detect such changes could be leveraged as part of a public health response.

## INTRODUCTION

Seasonal influenza remains a major public health burden. The constantly evolving virus infects ~10% of the global population each year during waves of generally unpredictable magnitude and timing (1). In 2021, widespread mitigation efforts against SARS-CoV-2 led to suppression of influenza circulation (2). In 2022, however, the uncertain impact of reductions in mitigation strategies created an opportunity to test the efficacy of wastewater-based surveillance (WBS).

Many viruses with public health significance are feasible targets for WBS (3). College campuses in particular are highly involved in these efforts due to essential close contact occurring during education and residential living (4). In preparation for influenza season, we converted our SARS-CoV-2 wastewater surveillance program into a broader public health effort. We aimed to detect the arrival and estimate the case magnitude of seasonal influenza in urban New York City college dormitory buildings (Table 1). We hoped to leverage longitudinal samples acquired over consecutive years to monitor influenza in wastewater at building-level resolution between seasons.

**TABLE 1:** Details of urban college buildings monitored in public health wastewater-based surveillance program

	Occupants (no.)^*a*^		Establishments (no.)^*b*^		Sampling schedule		
Building	Students	Nonstudents	Restaurants	Shops	Collection^*c*^	Time	*n*/h
Dormitory 1	136–138	1	0	0	Tu, F	0900^*d*^–0900	1
Dormitory 2	183–188	2	0	0	Tu, F	0900^*d*^–0900	1
Dormitory 3	188–191	~20^*e*^	2	1	Tu, F	2100^*d*^–0900	2

^*a*^ The number of student residents modestly differed between the fall 2021 and fall 2022 semesters.

^*b*^ Non-college-affiliated businesses that shared the same sewage outflow pipe.

^*c*^ Tu, F, Tuesday, Friday, respectively.

^*d*^ Sampling start time began day prior to collection.

^*e*^ Sixteen apartment units were presumed to contain one occupant, but the actual number may be larger.

## RESULTS

### WBS detection of seasonal influenza A

**Fig 1:**
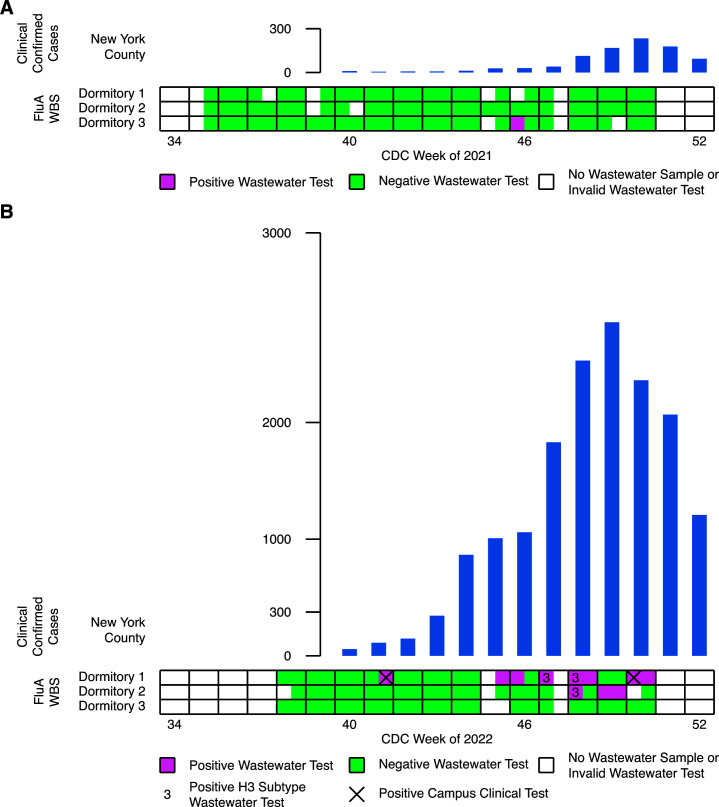
Wastewater-based surveillance of influenza A in dormitory building wastewater compared to local clinical case reports. Results for PCR testing of building wastewater from three dormitories during the (A) 2021 and (B) 2022 influenza seasons. Each box indicates a week consisting of two wastewater sampling dates. Positive and negative wastewater tests are depicted as reddish purple and bluish green squares, respectively. Invalid wastewater tests or the absence of sampling is depicted as a white square. A 3 denotes a positive H3 subtype PCR test. An X denotes a clinical confirmed case of a building resident. The height of the blue bars indicates the number of clinical confirmed cases reported in New York County for CDC weeks 40–52.

We detected very little influenza A in wastewater from the fall 2021 semester. Only 1 out of 79 valid samples collected during the Centers for Disease Control and Prevention (CDC) weeks 35–50 tested positive (Fig. 1A). The positive sample was obtained in November during week 46. Confirmed clinical cases in New York County increased from tens to hundreds in the same time frame (Fig. 1A).

Wastewater samples collected during the fall 2022 semester revealed a frequent and heterogenous occurrence of influenza A. A total of 11 out of 69 valid samples collected during weeks 39–50 tested positive (Fig. 1B). One building yielded a positive sample as early as October in week 41. Confirmed clinical cases in New York County increased from tens to hundreds in the same time frame (Fig. 1B). By December, two different buildings yielded 10 additional positive samples as confirmed clinical cases rose to thousands. Additional assays further identified a specific subtype in some samples. We confirmed the presence of H3 genomes on 3 days in November; we could not detect the presence of H1 genomes. It is noteworthy that positive samples were not evenly distributed among buildings. Dormitory 1 yielded more and earlier positive samples than other buildings; dormitory 3 yielded no positive samples. These differences occurred despite the similar number of occupants in those buildings (Table 1).

Positive wastewater tests in fall 2022 are associated with campus clinical data. Influenza incidence is generally underreported; only two cases were confirmed among residents of the three buildings studied. These cases, however, lived in dormitory 1, the building with the most positive wastewater results (Fig. 1B). Both patients were diagnosed on days that positive wastewater was also sampled.

The 2022 data stand in stark contrast to the 2021 results by revealing the more frequent and earlier presence of influenza A. The ~10-fold increase in positive wastewater samples mirrored the ~10-fold increase in New York County cases. This change in wastewater positivity is significant; a Fisher’s exact test rejects the null hypothesis that wastewater from 2021 and 2022 are equally likely to be positive (*P* < 0.01). Degradation during storage and freeze-thaw likely does not account for the difference as we could replicate ~70% of positive results from fall 2022 in 2023. In 2021, a positive wastewater sample was found in November when local clinical cases began to rise. In 2022, a similar number of local clinical cases appeared earlier, in October, and wastewater surveillance also detected a positive sample at that time.

### Quantitative comparison of wastewater testing and clinical cases

**Fig 2:**
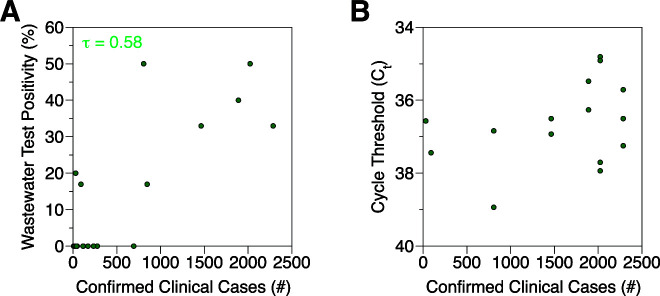
Quantitative assessment of the association between influenza A campus wastewater-based surveillance and local clinical case reports. Correlation between the number of clinical confirmed cases reported in New York County and (A) weekly wastewater test positivity or (B) C_t_ values of undiluted RNA. Data from 2021 and 2022 were combined. The correlation coefficient is shown as Kendall’s τ with a *P* value of <0.05.

Wastewater surveillance data quantitatively correlate with local confirmed clinical cases in terms of positivity rate but not cycle threshold (C_t_) values. Weekly wastewater test positivity strongly correlates with New York County clinical cases (Fig. 2A) as determined by a non-parametric ordinal association test (Kendall’s τ = 0.58). Individual C_t_ values, however, do not show a statistically significant association (*P* > 0.05) (Fig. 2B). Observed C_t_ values generally fall close to the limit of detection, where variability may be high.

### Absence of influenza B detection by WBS

**Fig 3:**
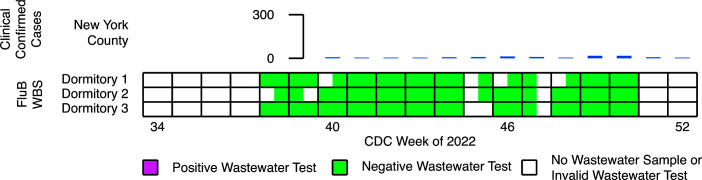
Wastewater-based surveillance of influenza B in dormitory building wastewater compared to clinical case reports. Results for PCR testing of building wastewater from three dormitories during the 2022 influenza season. Each box indicates a week consisting of two wastewater sampling dates. Positive and negative wastewater tests are depicted as reddish purple and bluish green squares, respectively. Invalid wastewater tests or the absence of sampling is depicted as a white square. The height of the blue bars indicates the number of clinical confirmed cases reported in New York County for CDC weeks 40–52.

We detected no influenza B in wastewater from the fall 2022 semester. All 66 valid samples collected during weeks 39–50 tested negative (Fig. 3). Confirmed clinical cases in New York County remained very low, not exceeding 17 per week, in the same time frame (Fig. 3). Campus clinical data matched those from New York County. Of the 23 students who tested positive for influenza during wastewater surveillance, all 23 were positive for influenza A and 0 were positive for influenza B.

### Influenza A fractionation and stability in wastewater

**Fig 4:**
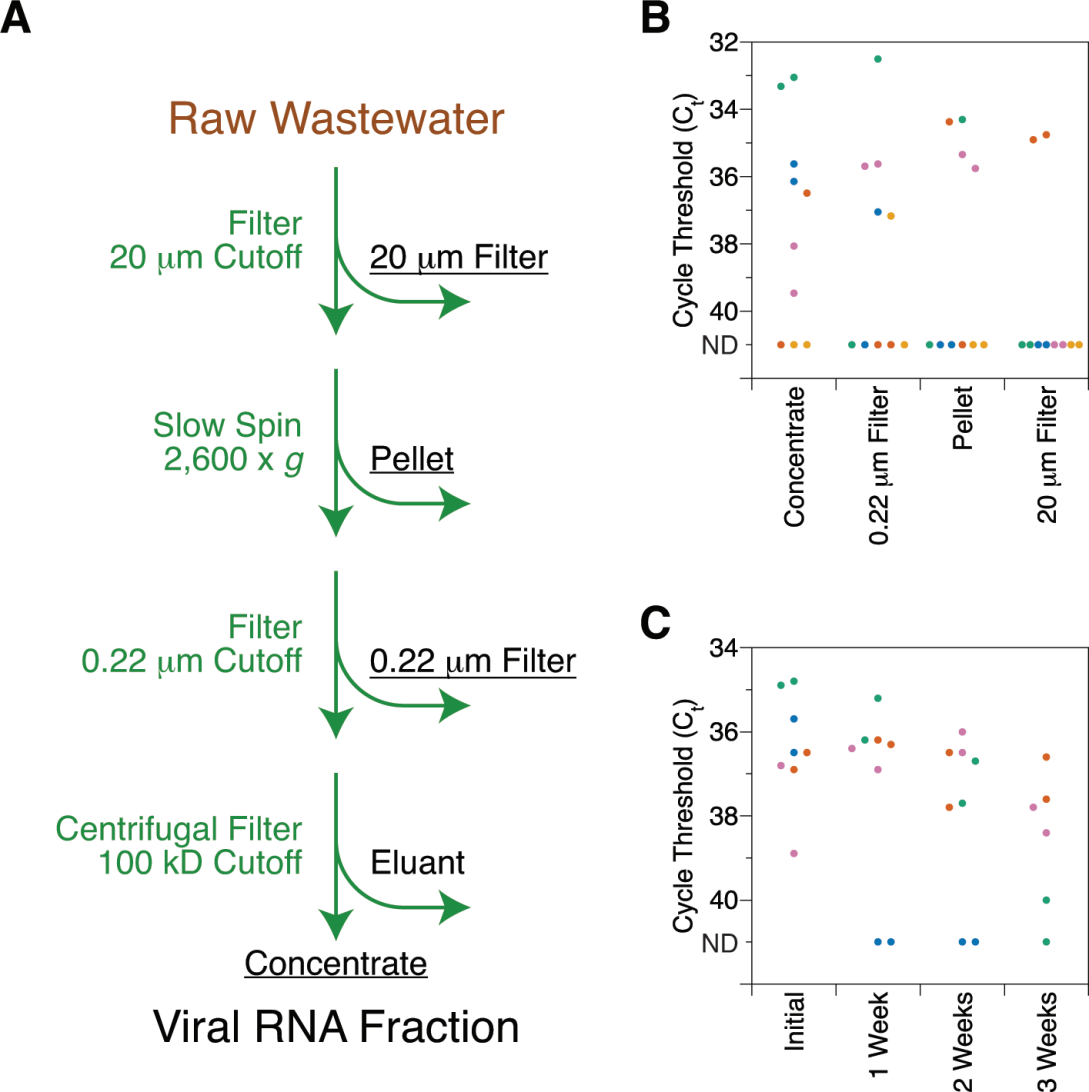
Partitioning and stability of influenza A in wastewater. (**A**) Schematic of accessible preparation of influenza A from wastewater for RNA analysis. Underlined labels indicate fractions subjected to RNA purification. (**B**) Quantitation of influenza A RNA purification from different fractions. C_t_ values of undiluted RNA were obtained in technical duplicate for each fraction. ND indicates not detected. Dot colors indicate *n* = 5 independent biological replicates. (**C**) Quantitation of influenza A RNA purification from the same sample over time. C_t_ values of undiluted RNA were obtained in technical duplicate for each time point. ND indicates not detected. A positive signal in the last 5 of the 45 PCR cycles is assigned a value of 40. Dot colors indicate *n* = 4 independent biological replicates.

We also systematically analyzed our viral purification protocol to identify in which fraction influenza can be found. Our accessible approach (5) includes sequential steps using a 20 µm filter, low-speed centrifugation, a 0.22 µm filter, and ultrafiltration to enrich for viruses (Fig. 4A). We can qualitatively argue that, while much virus can be found in solid fractions captured by filters or centrifugation, a detectable amount remains in the final concentrated liquid fraction (Fig. 4B). We further asked how long viral RNA in this liquid fraction remained detectable after storage in a refrigerator. Different samples behaved variably, with one still yielding RNA after 3 weeks but another losing detectable RNA after 1 week (Fig. 4C).

## DISCUSSION

### Assessment of seasonal influenza WBS

WBS can quantitatively measure the magnitude and timing of seasonal influenza A between years. First, the number of positive wastewater samples on campus followed a similar magnitude increase as clinical reports in the local New York County area. Second, the temporal timing of detection in wastewater also matched the earlier shift in clinical reports. Wastewater test positivity quantitatively correlates with local clinical cases.

Our surveillance could not detect influenza B. Our study focused on influenza A given the relatively low incidence of influenza B infection immediately after 2020 (6). Influenza B may, however, be underreported in clinical cases and still appear at levels comparable to influenza A in wastewater (7). This underreporting has not been consistently observed (8), necessitating more studies. Similarly, we also could not observe this discordance. We attribute the lack of wastewater signal to the low number of clinical cases both on campus and in New York County.

### Experimental considerations for wastewater processing

Our ability to detect influenza A in the liquid phase of wastewater contrasts with initial work from the field and is a counterpoint useful in planning future experiments. Previous studies detected almost all influenza in solid fractions that could be trapped by filters or high-speed centrifugation (9, 10). Our ultrafiltration protocol consisting of filtration and low-speed centrifugation (5) did not yield similar results. We were instead able to obtain influenza from the liquid phase as well as from different solid fractions.

### Impact of influenza WBS

Building-level wastewater surveillance localized the heterogeneity of influenza variation during the large 2022 seasonal surge. Our work focuses on individual buildings as opposed to larger sewersheds studied by others (7–10). Some but not all buildings may display interseasonal influenza variation of magnitude and timing. The ability to detect localized changes could be leveraged as part of a public health response. Occupants of specific buildings may be more receptive to communication and outreach if presented with location-specific data. Information about the presence of influenza on campus can be shared both with providers, to increase their index of suspicion for influenza, as well as with the larger college community, to encourage mitigation strategies such as mask use, handwashing, and avoidance of community events when symptomatic. Quantitative understanding of the timing and magnitude of seasonal variation may also guide vaccination recommendations and strategies. Other settings with vulnerable populations, such as nursing homes, daycares, and cancer centers, could further benefit from knowledge of elevated circulating influenza levels. Our work contributes to a growing literature highlighting the potential impact of building-level WBS.

## MATERIALS AND METHODS

### Wastewater sampling and processing

Our wastewater surveillance program (11) measured viral RNA in the sewage outflow of three dormitories at Barnard College twice a week in 2021 and 2022 (Table 1). An autosampler collected 12- or 24-hour composite samples into a refrigerated container at 4°C. Technical difficulties sometimes but infrequently resulted in grab or incomplete composite samples. The heat-inactivated liquid fractions of samples were enriched for viruses using our accessible ultrafiltration protocol (5).

To measure the fractionation and stability of influenza A in wastewater, we repeated testing of positive samples stored at 4°C after heat inactivation. For fractionation experiments, three samples were reprocessed within a week after collection and two were reprocessed within a month after collection. RNA was purified using the Quick-RNA Miniprep Kit (https://www.zymoresearch.com). Cut filters, pellets, and concentrated liquids were mixed directly with RNA Lysis Buffer (https://www.zymoresearch.com). For stability experiments, we reprocessed positive samples after 1, 2, and 3 weeks.

### Diagnostic testing

We modified our SARS-CoV-2 RT-qPCR protocol (5) for this study. To detect influenza, we substituted in previously validated concentrations of the InfA and InfB primers and probes (12) (https://www.idtdna.com/). These consisted of the following sequences: INFA Forward 1 Primer CAAGACCAATCYTGTCACCTCTGAC, INFA Forward 2 Primer CAAGACCAATYCTGTCACCTYTGAC, INFA Reverse 1 Primer GCATTYTGGACAAAVCGTCTACG, INFA Reverse 2 Primer GCATTTTGGATAAAGCGTCTACG, INFA (FAM) Probe (FAM)TGCAGTCCT(ZEN)CGCTCACTGGGCACG(3IABkFQ), INFB Forward Primer TCCTCAAYTCACTCTTCGAGCG, INFB Reverse Primer CGGTGCTCTTGACCAAATTGG, and INFB (YAK) Probe (YakYel)CCAATTCGA(ZEN)GCAGCTGAAACTGCGGTG(3IABkFQ). We reproduced singleplex specificity for influenza A and B by performing standard curves with genomic RNA from influenza A virus, A/Puerto Rico/8/1934 (H1N1), NR-2773 (https://www.beiresources.org/), and genomic RNA from influenza B virus, B/Texas/06/2011 (Yamagata Lineage), NR-45849 (https://www.beiresources.org/).

To subtype influenza, we substituted in previously validated concentrations of the NIID-swH1 and NIID-H3 primers and probes (13) (https://www.idtdna.com/) (https://www.thermofisher.com/). These consisted of the following sequences: NIID-swH1 Taqman Primer-F1 AGAAAAGAATGTAACAGTAACACACTCTGT, NIID-swH1 Taqman Primer-R1 TGTTTCCACAATGTARGACCAT, NIID-swH1 Probe2 (FAM)CAGCCAGCAATRTTRCATTTACC(MGB), NIID-H3 Taqman Primer-F1 CTATTGGACAATAGTAAAACCGGGRGA, NIID-H3 Taqman Primer-R1 GTCATTGGGRATGCTTCCATTTGG, and NIID-H3 Probe1 (FAM)AAGTAACCCCKAGGAGCAATTAG(MGB).

RNA purified from dormitories during the fall 2021 semester was assayed in 2022 and 2023 from archives stored at −80°C. Most RNA purified from dormitories during the fall 2022 semester was assayed on the same day with exceptions assayed in 2023 from archives. Invalid results with no signal from the human RP primer set were discarded.

### Clinical data

Students who visited the on-campus health service for laboratory testing during wastewater surveillance were administered an Influenza A & B Test (https://mms.mckesson.com/). Influenza-positive laboratory results for New York County were downloaded from the New York State Department of Health (14).
